# The future of scientific periodicals: A librarian's perspective

**DOI:** 10.1098/rsnr.2016.0038

**Published:** 2016-08-24

**Authors:** Stella Butler

**Affiliations:** The Brotherton Library, University of Leeds, Leeds LS2 9JT, UK

Over the past two decades, libraries have moved from being warehouses of print journals to gateways to the electronic scholarly record. ‘Gate*keepers*’ is perhaps a more accurate term as universities no longer own journals. Instead we buy licences which allow only university students and staff to read these digital texts. This has undoubtedly created legal barriers to access for many outside the academy who previously could access print journals by visiting a library and, where appropriate, using the photocopier. This new situation seems counter-intuitive to what might have been expected from a move to the openness of the web-based digital environment that we all now inhabit.

Scholars in science and medicine were the first to react to these barriers by establishing ‘open access’ journals. Some of these journals advocated ‘open evaluation’ using the potential of the web for ‘crowd-sourcing’ reviews and opinions of articles, taking the periodical way beyond what is possible in the print realm, reinforcing the notion of community but also extending it beyond the academy. This changed operating model found resonance with major research funders who wanted research to be widely and quickly disseminated. They began to apply the principle that the outputs of publicly funded research should be available to all. Traditional publishers reacted by offering pay-to-publish opportunities in subscription journals, giving birth to the so-called hybrid journals and conversations between publishers and libraries about ‘double-dipping’. Publishers have also allowed articles in subscription journals to be made open access by deposit in either subject or institutional repositories. This has become known as the ‘green’ route to open access.

The administration of these complex payment arrangements (which sometimes involve page charges for tables or colour or both) and issues around funder-compliance have brought enormous challenges to the administration of the scholarly record for both researcher and library.

The fluidity of the digital environment also raises issues of preservation and stability. Journals are now bytes in the cloud—bytes belonging to publishing organizations which, as history tells us, rise and fall over time. So what can publishers guarantee in terms of perpetual access? The seventeenth-century publications of the Royal Society are still as ‘bright’ and clean as when they first came off the press. In contrast, the digital modern *Domesday Book* produced by the British Broadcasting Corporation (BBC) in 1986 was almost lost by the year 2000 and required enormous investment to unlock the information contained on the laser discs. Both publishers and the library community are undoubtedly getting better at digital preservation. But whereas I can say for sure that given good storage conditions you will be able to continue to read our printed books for centuries to come, I am not sure that we yet have the same control over these changing entities that are coming to form our scholarly output. And if in the future we do lose access to journals how can we continue to benefit from twenty-first-century scholarship?

